# The Fight Against Severe COVID-19: Can Parasitic Worms Contribute?

**DOI:** 10.3389/fimmu.2022.849465

**Published:** 2022-02-11

**Authors:** Pengfei Cai, Yi Mu, Donald P. McManus

**Affiliations:** Molecular Parasitology Laboratory, QIMR Berghofer Medical Research Institute, Brisbane, QLD, Australia

**Keywords:** COVID-19, SARS-CoV-2, cytokine storm, viral sepsis, helminth, immunomodulatory therapy

## Introduction

As of 31 December 2021, COVID-19, caused by infection with SARS-CoV-2, had been confirmed in more than 285 million people worldwide, with more than 5.4 million dead resulting in a case fatality ratio of 1.89%. This figure is likely to be vastly underestimated, as a proportion was not registered officially as COVID-19-related/excess deaths. The United States recorded the highest number (54,656,866) of confirmed cases. In Africa, there are 47 countries affected, with 7,065,972 cumulative cases and 155,081 deaths were recorded by 31 Dec 2021 (WHO African Region numbers at a glance). To date, the currently approved vaccines have been effective in preventing COVID-19, particularly in regards to severe symptoms (1). However, several immune escape mechanisms of SARS-CoV-2 and the rapid emergence of mutated variants (2) pose a great challenge to the efficacy of these vaccines.

Patients with severe COVID-19 tend to have a high concentration of pro-inflammatory cytokines (IL-2, IL-7, IL-10, G-CSF, TNF-α, CXCL10, MCP1, and MIP1α) (3), suggesting that a cytokine release syndrome (CRS) (4) (also loosely referred to as a cytokine storm), which is a form of life-threatening systemic inflammatory response syndrome (SIRS), can often feature in severe COVID-19 infections. Among the increased levels of inflammatory mediators in COVID-19 patients, the plasma levels of IL-6, an amplifier in the cytokine storm, are significantly elevated in non-survivors compared with survivors (5). The main cause of death of COVID-19 is due to severe acute respiratory distress syndrome (ARDS) with this high severity being dependent on the cytokine storm.

Sepsis has been defined as a life-threatening organ dysfunction caused by a dysregulated host response to infection (6). Endothelium damage, vascular permeability, microvascular dysfunction, coagulation pathway activation, and impaired tissue oxygenation occur during sepsis and can lead to multisystem organ dysfunction (MODS), organ failure and consequently a potentially lethal outcome. As many patients with severe COVID-19 show typical clinical manifestations of septic shock, with other symptoms meeting the diagnostic criteria for sepsis and septic shock according to the Sepsis-3 International Consensus (6), Li et al. hypothesized that viral sepsis is a crucial process in severe COVID-19 cases (7). Accumulating evidence further links the pathology of severe COVID-19, such as acute kidney injury, to sepsis (8).

## Immunomodulatory Therapy of Severe COVID-19

In regards to potential immunomodulatory strategies for severe COVID-19, the IL-6-STAT3 signaling pathway has been considered a promising therapeutic target for the cytokine storm generated in the disease. Tocilizumab, a specific monoclonal antibody that blocks IL-6, has been recommended for use in critically ill COVID-19 patients with extensive bilateral pulmonary lesions and with elevated serum levels of IL-6. However, anti-cytokine therapy with Tocilizumab did not improve survival rates despite reducing the likelihood of progression to the composite outcome of mechanical ventilation or death (9). In addition, ulinastatin, a serine protease inhibitor with anti-inflammatory properties (including inhibition of IL-6), previously used in the treatment of acute pancreatitis and sepsis, has been suggested for severe COVID-19 treatment (10); yet its clinical performance and cost-effectiveness remain to be validated in large cohort studies.

The value of glucocorticoids in mitigating the inflammatory response due to COVID-19 has been widely scrutinized. Recent reliable evidence from large-scale randomized clinical trials (RCTs) revealed that the use of dexamethasone reduced 28-day mortality but only in patients requiring respiratory support (11), while another parallel, double-blind, placebo-controlled, randomized, Phase IIb clinical trial showed that the administration of methylprednisolone was able to reduce 28-day mortality in patients aged over 60 years (12). In addition, hydroxychloroquine, a disease-modifying antirheumatic drug (DMARD), used for the treatment of rheumatoid arthritis and lupus, has been studied for its potential as an immunomodulatory therapeutic for COVID-19 disease. Evidence from 12 RCTs indicated that hydroxychloroquine has little or no effect on the risk of death, probably has no effect on progression to mechanical ventilation, and that it is less likely that the drug is effective in protecting people from infection, although this was not excluded entirely (13). Other immunomodulatory agents that have been therapeutically tested in SARS-CoV-2 infection include the interleukin-1 receptor (IL-1R) antagonist anakinra, the Janus kinase inhibitors baricitinib and ruxolitinib, the anti-C5a antibody vilobelimab, the anti-gout agent colchicine, the antirheumatic drug leflunomide, convalescent plasma, interferon beta, interferon kappa and intravenous immunoglobulins (IVIg) (14). However, robust data from further RCTs are required to elucidate their potential for the treatment of severe COVID-19.

## Helminth Co-Infection and Severity of COVID-19

The “old friends” hypothesis argues that some co-evolved microbes and other pathogens, including helminths, could help to establish appropriate immunomodulatory function and thus protect the host against a large spectrum of immune-related disorders (15). Mammals infected with helminths typically elicit an anti-inflammatory Th2 immune response, including the activation of Th2 cells and the elevation of Th2-type cytokines such as IL-4, IL-5 and IL-13 (16). This host-helminth interaction could be beneficial in dampening inflammatory damage induced by the Th1/Th17 branches of the immune system, repairing injured tissue and restoring homeostasis (17). Chronic helminthic infection suppresses both Th1 and Th2 responses by actively inducing the expansion of FOXP^3+^ regulatory T cells, IL-10 producing B cells and alternatively activated macrophages (AAMs), which together promote the release of regulatory cytokines such as TGF-β and IL-10 (18).

There is controversy regarding whether helminth coinfection leads to increased susceptibility and attenuated immunopathology of other pathogens (i.e., viruses, bacteria and protozoa) or, in some circumstance, exacerbated pathology due to higher infection burdens (19). And this also likely applies to the interaction between helminths and SARS-CoV-2 (20, 21). It has been suggested that the immunosuppressive and regulatory T-helper response stimulated by helminths may balance the inflammatory Th1/Th17 response triggered by SARS-CoV-2 infection, potentially restricting the severity of COVID-19 disease (22, 23). In contrast, a recent viewpoint article argued that COVID-19 patients co-infected with helminths may be unable to mount a quick and efficient immune response against SARS-CoV-2 in the early phase of the infection, thereby leading to increased patient morbidity and mortality (24). However, other evidence indicates that COVID-19 lethality rates are significantly lower in Sub-Saharan Africa than in the industrialized world (25). Wolday et al. (26) carried out a prospective observational cohort study to investigate whether there was a potential correlation between co-infection with intestinal parasites and the severity of COVID-19 in two sites in an endemic area of Ethiopia in Sub-Saharan Africa. The study revealed that patients co-infected with parasites had lower odds of developing severe COVID-19, with an adjusted odds ratio (aOR) of 0.23 (*p* < 0.0001) for all parasites, an aOR of 0.37 (*p* < 0.0001) for protozoa, and an aOR of 0.26 (*p* < 0.0001) for helminths. The authors thus concluded that co-infection with the enteric parasites, *Hymenolopis nana*, *Schistosoma mansoni* and *Trichuris trichiura* reduced the risk of severe COVID-19 occurrence in this cohort of African patients. When stratified by species, co-infection with *T. trichiura* showed the lowest probability of developing severe COVID-19. In addition, of 11 cohort patients who died, all were parasite-free (26). The results of this study thus suggested that parasites, particularly the chronic disease-associated parasitic helminths, induced a Th2-prone response in the host, which modulates COVID-19 severity by restricting the hyper-inflammation associated with the viral infection. Further epidemiological studies on helminth-mediated COVID-19 alleviation are, however, required to support this argument (27, 28). 

## Helminth-Derived Products Can Attenuate the Severity of Sepsis

The “old friends” hypothesis, together with the inverse global distribution of allergy/autoimmune diseases and helminth infections, and the proclivity for helminths to orchestrate immunomodulatory effects (typically induction of a Th2 immune response) on the host immune system stimulated the concept of developing helminth-based therapies. Robust evidence from animal model studies showed that helminth infection and helminth-derived products were able to prevent/alleviate a variety of autoimmune and inflammatory diseases/disorders (i.e., sepsis, type 2 diabetes, allergic asthma, rheumatoid arthritis, inflammatory bowel disease, type 1 diabetes and multiple sclerosis) (29, 30).

In regard to sepsis, epidemiological studies (over the period 2006-2015) indicated a rapid increase in hospitalization and mortality rates due to severe sepsis in high-income countries (31). This report added further support to the hypothesis that the lack of helminth infections may contribute to the aetiology of sepsis (32). To date, a number of helminth-derived molecular products have resulted in improved sepsis outcomes in animal models. Several studies have investigated the role of *Schistosoma japonicum* cystatin (rSj-Cys) in regulating the inflammatory response in the cecal ligation and puncture (CLP)-induced mouse sepsis model (33–35). Administration of rSj-Cys to mice provided significant therapeutic effects on CLP-induced sepsis characterized by increased survival rates, alleviated overall disease severity with reduced tissue injury in the kidney, lung and liver (33) and cardiomyopathy (34). These therapeutic effects were linked to the upregulation of regulatory cytokines (IL-10 and TGF-β1) and the downregulation of pro-inflammatory cytokines (IL-1β, IL-6 and TNF-α) as measured in serum. Similarly, treatment of mice with cyclophilin A (CsCyPA) from the liver fluke, *Clonorchis sinensis*, provided significant therapeutic effects on CLP-induced sepsis characterized by an improved survival rate (36). Furthermore, using a murine model of septic shock, Ramos-Benitez et al. demonstrated *in vitro* and *in vivo* that Fh15, a recombinant variant of the common liver fluke *Fasciola hepatica* fatty acid binding protein, suppressed the LPS-induced cytokine storm, working as an antagonist of Toll-like receptor 4 (TLR4) (37). In the gram-negative bacteria-induced sepsis rhesus macaque model, Fh15 effectively suppressed bacteremia, endotoxemia, and many other inflammatory markers, emphasizing its promise as a candidate for immunomodulatory therapy against sepsis (38). In addition, the excretory-secretory products of *Trichinella spiralis* adult worms were also shown to be beneficial to the outcome of CLP-induced sepsis by preventing exacerbated inflammation and severe pathology in treated mice (39). These effects were associated with reduced levels of pro-inflammatory cytokines (IL-1β, IL-6 and TNF-α), upregulated levels of IL-10 and TGF-β, and decreased expression of HMGB1, TLR2 and MyD88 in the lung tissues of the treated mice (39). Albeit informative, these observations were accrued from animal sepsis models and human clinical trials now need to be undertaken to validate the results obtained. 

## Discussion

Rapid mutations in SARS-CoV-2 challenge the efficacy of the current COVID-19 vaccines and concerns about their long-term safety require an urgent need to search for safe and cost-effective alternatives for preventing severe COVID-19 disease. Increased efforts are required as, to date, conclusive evidence of effective immunomodulatory therapies for severe COVID-19 is scarce (14). Due to similarities in the pathological process, sepsis animal models provide the opportunity to evaluate the efficacy of novel candidates for the immunomodulatory therapy of critically ill COVID-19 patients. We argue that helminth-derived products and molecules that can potentially induce a Th2-biased immune response may provide a contributory role in preventing severe COVID-19 by restricting the cytokine storm associated with ARDS. The aforementioned helminth-derived molecules (i.e. rSj-Cys, CsCyPA and Fh15) have been shown to increase survival rates in animal models of sepsis, thereby representing potential candidates for immunomodulatory treatment against severe COVID-19. Such components should be validated for efficacy, first in the K18-hACE2 transgenic murine model of SARS-CoV-2 infection which shares many features of severe COVID-19 infection (40), and then in clinical cohorts. The time phase in sepsis progression is regarded as a key factor for successful immunomodulatory therapy. Due to immunosuppression and immune exhaustion, treatment with immunomodulators at the late stage of severe COVID-19 could be less effective or even deleterious; consequently, the helminth-derived molecular products should be administrated as a prophylactic therapy against severe COVID-19. The suppression of the antiviral response due to excessive immunotherapy may encourage viral replication and result in a delay of clearance of SARS-CoV-2 so that administration of helminth-derived immunomodulators that elicit a mild Th2-skewed immune response could be a useful strategy to prevent severe COVID-19, while maintaining the patient’s ability to kill cells infected with the virus. Severe COVID-19 has greater incidence in older individuals, due in part to an increased inflammatory response in these patients (41), begging the question whether prophylactic therapy based on helminth-derived product should primarily target and would be more effective in older individuals? Another unanswered question is whether a well-controlled low level concomitant infection with a live helminth, such as hookworm (42), can achieve an equivalent or superior effect to an immunomodulator or vaccine in preventing serious outcomes of COVID-19. It would be valuable to test such helminthic-based therapies as these may represent a safe and cost-effective anti-inflammation approach to reducing COVID-19 severity.

## Author Contributions

Conceptualization: PC, YM, and DM. Writing – original draft: PC and YM. Writing – review and editing: PC and DM. All authors contributed to the article and approved the submitted version.

## Funding

This work was partly supported by the National Health and Medical Research Council (NHMRC) of Australia (ID: APP1160046, APP2008433 and APP1098244). DPM is a NHMRC Leadership Fellow and Senior Scientist at QIMRB.

## Conflict of Interest

The authors declare that the research was conducted in the absence of any commercial or financial relationships that could be construed as a potential conflict of interest.

## Publisher’s Note

All claims expressed in this article are solely those of the authors and do not necessarily represent those of their affiliated organizations, or those of the publisher, the editors and the reviewers. Any product that may be evaluated in this article, or claim that may be made by its manufacturer, is not guaranteed or endorsed by the publisher.
